# Novel Applications of NSAIDs: Insight and Future Perspectives in Cardiovascular, Neurodegenerative, Diabetes and Cancer Disease Therapy

**DOI:** 10.3390/ijms22126637

**Published:** 2021-06-21

**Authors:** Edmundas Kaduševičius

**Affiliations:** Institute of Physiology and Pharmacology, Medical Academy, Lithuanian University of Health Sciences, 9 A. Mickeviciaus Street, LT-44307 Kaunas, Lithuania; edmundas.kadusevicius@lsmuni.lt

**Keywords:** NSAIDs, neuro-inflammation, axonal damage, demyelination, atherosclerosis, carcinogenesis, diabetes

## Abstract

Once it became clear that inflammation takes place in the modulation of different degenerative disease including neurodegenerative, cardiovascular, diabetes and cancer the researchers has started intensive programs evaluating potential role of non-steroidal anti-inflammatory drugs (NSAIDs) in the prevention or therapy of these diseases. This review discusses the novel mechanism of action of NSAIDs and its potential use in the pharmacotherapy of neurodegenerative, cardiovascular, diabetes and cancer diseases. Many different molecular and cellular factors which are not yet fully understood play an important role in the pathogenesis of inflammation, axonal damage, demyelination, atherosclerosis, carcinogenesis thus further NSAID studies for a new potential indications based on precise pharmacotherapy model are warranted since NSAIDs are a heterogeneous group of medicines with relative different pharmacokinetics and pharmacodynamics profiles. Hopefully the new data from studies will fill in the gap between experimental and clinical results and translate our knowledge into successful disease therapy.

## 1. Introduction

Historically, medicines applied for the relief of pain, fever, and inflammation from herbs or plants were known for centuries. The first record was about 3500 years ago in the Ebers papyrus. Hippocrates, Celsus, Pliny the Elder, Dioscorides, and Galen recommended decoctions containing salicylate for rheumatic pain [1]. Edward Stone made most probably the first “clinical trial” and found that 1 dram (1.8 g) of willow bark reduced fever in 50 patients [2]. Felix Hoffmann, a chemist of Friedrich Bayer & Co in Elberfeld laboratory, prepared the first sample of pure acetylsalicylic acid on 10 August 1897. Bayer’s Research Director Dr. Heinrich Dreser tested acetylsalicylic acid in animals, found antipyretic, analgesic, and anti-inflammatory properties, and marketed the product in 1899 under the trademark of Aspirin [3]. Neither tablets nor sugar-coating tablets were available at that time, thus aspirin was introduced on market in powder pharmaceutical form and had a bitter taste [1]. Later on, in Germany, a few antipyretic/analgesic agents likeantipyrine, aminopyrine, phenacetin, and paracetamol (acetaminophen) as the active metabolite of phenacetin were commercially developed for use in the management of pain, fever, and inflammation in the 1950s [4].

It looks like the chemical advances of the 19th–20th centuries promoted the development of non-steroidal anti-inflammatory drugs (NSAIDs), most of which were initially organic acids, and later on non-acidic compounds were discovered [4]. It was agreed that aspirin was recognized as the progenitor of the pharmacotherapeutic class of NSAIDs medicines and the first in the class of medicine was phenylbutazone in 1946 and indomethacin in the 1960s [5] continuing with other NSAIDs, including ibuprofen, diclofenac, naproxen, piroxicam [6]. The progress of new NSAIDs development and introduction to the market was derived by the discovery of the mechanism of action NSAIDs drugs based on the inhibition synthesis of prostaglandins via an arachidonic acid pathway. In 1971, Sir Vane demonstrated that aspirin and NSAID related drugs inhibit the formation of prostaglandins associated with pain, fever, and inflammation [7], thus providing a physiologic rationale for the use of NSAIDs in the management of pain, fever, and inflammation.

The British pharmacologist John Robert Vane shared the 1982 Nobel Prize in physiology or medicine with Swedish scientists Sune K. Bergström and Bengt I. Samuelsson for their discoveries concerning prostaglandins and related biologically active substances that influence blood pressure, body temperature, allergic reactions, and other physiologic phenomena in mammals [8,9] and NSAIDs have become first choice drugs for the treatment of various pain, fever, and inflammation conditions.

## 2. Mechanism of Action and Classification of NSAIDs

Despite Sir Vane’s prostaglandin hypothesis has been generally accepted, various in vitro studies have suggested that additional mechanisms may have a role in the effects of NSAIDs [10]. There have been many hypothesis and studies to link the anti-inflammatory action of so-called “anti-defensive or aspirin like” [11] medicines to their ability to inhibit the activity of endogenous substances like kinines [11,12] slow-reacting substance in anaphylaxis (SRS-A) [13] adenosine triphosphate (ATP) [14,15,16] arachidonic acid (AA) and prostaglandin F2α (PGF2α) [17,18]. In the 1990s an important discovery was made from molecular and cellular studies that there are two cyclooxygenase (COX) enzymes controlling the production of prostaglandins (PGs) and thromboxane A2 (TxA_2_). COX-1 enzyme that produces PGs and TxA_2_ that regulate gastrointestinal, renal, vascular, and other physiological functions, and COX-2 that regulates production of PGs involved in inflammation, pain, and fever [19,20].

Intention to avoid the gastrointestinal side effects associated with COX-1 inhibition stimulated the development of selective COX-2 inhibitors so-called “coxibs”, which were designed to inhibit COX-2 without altering COX-1 activity at therapeutic doses [21]. COX-2 inhibitors reduce inflammation without the risk of ulceration. The hypothesis of ideal NSAIDs with inhibition of COX-2 synthesis while preserving COX-1 [22] was set in the 1990s for the discovery and development of NSAIDs selective COX-2 inhibitors without effect on COX-1 [23] whose inhibition was being a major factor in the development of gastrointestinal adverse drug reactions of NSAIDs [24,25,26,27]. The discovery of two COX isoforms has triggered a rapid development of COX-2 selective inhibitors and very soon a new generation of NSAIDs so-called “COX-II inhibitors” like celecoxib, etoricoxib, rofecoxib, and others were placed to market.

For a long time, NSAIDs have been classified according to their chemical structure into two groups: NSAIDs of acidic and non-acidic origin. NSAIDs of acidic origin are subdivided according to the name of the organic acid which forms the basis of the structure of the medicinal product [28]. The differences between these drugs are small, but they can sometimes be relevant to clinical practice in the presence of hypersensitivity to NSAIDs [29].

Identification of COX isoenzyme selectivity has stimulated NSAIDs classification according to their relative inhibitory activities against COX-1 and COX-2. NSAIDs with the IC50 ratio (COX-2 IC50/COX-1 IC50) > 5 were classified as COX-1-selective inhibitors, and those with the IC50 ratio < 0.2 were classified as COX-2-selective inhibitors. NSAIDs classification according to selectivity to COX-1 and COX-2 is as follows: (1) COX-1 selective inhibitors (low-dosage aspirin); (2) COX non-selective inhibitors (the majority of classified NSAIDs, which when administered over the long term, e.g., in cases of rheumatoid arthritis, cause duodenal ulcers in 20% of cases and gastric hemorrhage in 1–4% of cases/year); (3) COX-2 preferential inhibitors (meloxicam and nimesulide, which have fewer gastric side effects than standard NSAIDs, but which are not risk-free at high doses); (4) COX-2 selective inhibitors (celecoxib and rofecoxib) [20].

**Figure 1:**
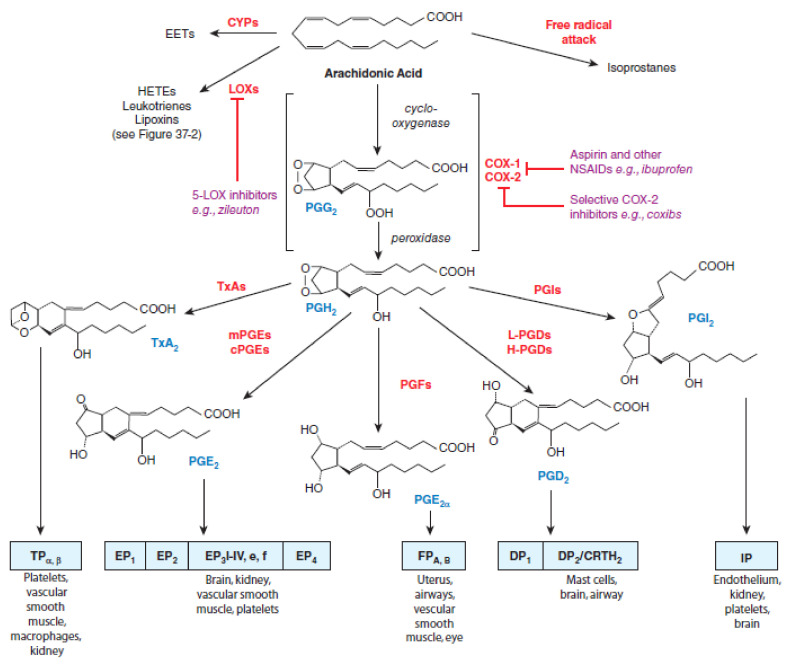
Cyclooxygenases pathways of arachidonic acid (AA) metabolism. With permission of Mc Graw Hill.

There are two isoforms of COX enzymes: the constitutively expressed COX-1 isoform and the inducible COX-2 isoform. COX-1 is present in the majority of cells and tissues, including the endothelium, monocytes, gastrointestinal epithelial cells, and platelets, while COX-2 is constitutively expressed in only a few tissues [30]. Expression of COX-2 is upregulated in a variety of cells and tissues, such as vascular endothelium, rheumatoid synovial endothelial cells, monocytes, and macrophages, during inflammation through the actions of various inflammatory mediators and the increase in COX-2 protein levels is the primary driving force for enhanced production of prostanoids at inflammatory sites [30,31]. Evidence from non-clinical trials suggests that COX-1 might play an important role in the contribution of the initial phase of prostanoid-dependent pain and inflammation [19,32]. The AA metabolism via cyclooxygenases pathway and the roles of COX-1 and COX-2 in different conditions are summarized in Figure 1.

**Figure 2:**
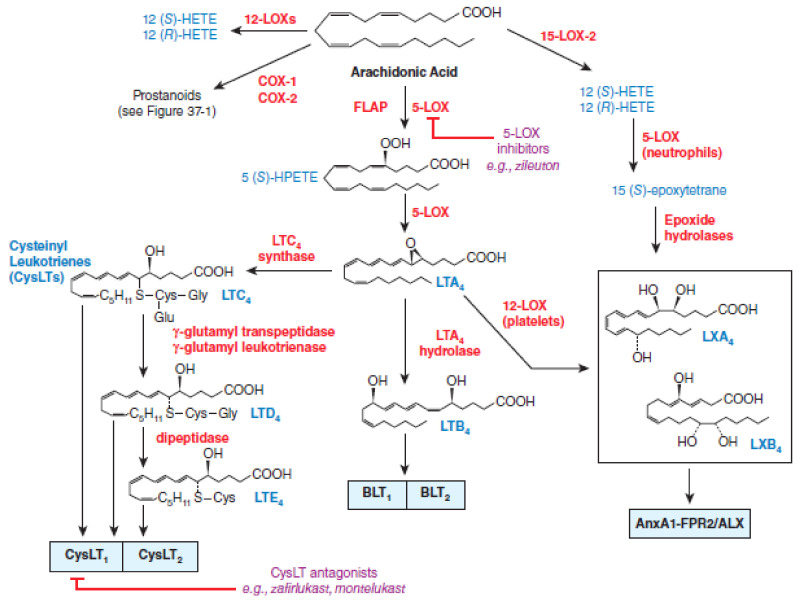
Lipoxygenase pathways of AA metabolism. With permission of Mc Graw Hill.

At the very beginning, the researchers focused investigations on the evaluation of the effects of NSAIDs on cyclooxygenase metabolism of arachidonic acid, but subsequent studies have shown that metabolites of the arachidonate 5-lipoxygenase (5-LOX) pathway “pro-inflammatory” cytokines such as tumor necrosis factor-alpha (TNF-α) and interleukin-1 (IL-1) may also play an important role in the modulation of various disorders since cytokines increase microvascular permeability and are potent chemotactic agents and attract eosinophils, neutrophils, and monocytes into the synovium [33,34]. Arachidonate 5-lipoxygenase pathway metabolites eicosanoids regulate a number of functions in T cells, including proliferation, apoptosis, cytokine secretion, differentiation, chemotaxis, and a wide array of physiological processes, starting from inflammatory processes such as asthma and allergies, to diseases such as cancer and AIDS [35]. The development of a new NSAIDs potential able to inhibit both cyclooxygenases and arachidonate 5-lipoxygenase have been started for a new therapeutic applications [36,37,38,39,40]. The dual-acting NSAIDs with dual inhibition of both COX and 5-LOX demonstrated neuroprotective effects by suppressing toxic actions of reactive microglia and macrophages, which are increased in the aging brain and in age-related degenerative conditions, such as Alzheimer’s and Parkinson’s diseases [36,37,41]. The dual-acting NSAIDs by blocking the 5-LOX pathway does not alter the synthesis of lipoxins (LXs), which are produced by lipoxygenation of 15(S)-hydroperoxyeicosatetraenoic acid (15(S)-HpETE), and which can have potent anti-inflammatory properties and can be considered as “stop-signal” mediators [36]. The lipoxygenase pathways of AA metabolism is presented in Figure 2.

The discovery of the COX isoforms and NSAIDs inhibition of leukotriene pathway of AA metabolism stipulated the hypothesis, that dual-acting NSAIDs may play an important role in non-arthritic or non-pain conditions where there is an inflammatory component to pathogenesis, including cancer, Alzheimer’s, and other neurodegenerative diseases. Non-clinical studies have supported evidence, that potential molecular (cyclooxygenases, secretases, nuclear factor kappa-light-chain-enhancer of activated B cells (NF-κB), peroxisome proliferator-activated receptors gamma (PPAR-γ), or a large family of hydrolase enzymes that bind to the nucleotide guanosine triphosphate ((Rho-GTPases) and cellular (neurons, microglia, astrocytes or endothelial cells) targets of NSAIDs may mediate the therapeutic function of NSAIDs in neurodegeneration [41,42].

Evidence from non-clinical trials indicates that eicosanoids and lipid mediators may are involved in cancer development surrounding inflammatory and stromal cell responses [43] and provides a reference for the potential benefits of NSAIDs in cancer chemotherapy via activation apoptosis [44] and modulation tumor autophagy through the *PI3K/Akt/mTOR, MAPK/ERK1/2, P53/DRAM, AMPK/mTOR, Bip/GRP78, CHOP/GADD153*, and *HGF/MET* signaling pathways and inhibition lysosome function, leading to p53-dependent G1 cell-cycle arrest [45].

Data from new preclinical studies, scientific and technological developments in the 21st century have stimulated research and clinical trials of NSAIDs, which have been commonly used to control inflammation, pain, and fever over the last few centuries, for new NSAIDs therapeutic targets never used before, including neurodegenerative disorders, psychiatric, epilepsy, cardiovascular, diabetes and cancer [43,46,47,48,49,50].

## 3. Neurodegenerative Diseases

### 3.1. Alzheimer’s Disease

Several studies have been conducted to evaluate the effects of NSAIDs on neurodegenerative diseases such as Alzheimer’s disease, multiple sclerosis, Parkinson’s disease, and amyotrophic lateral sclerosis since reports have identified the inflammatory process in the pathogenesis of neurodegenerative disorders [51]. Inflammation in the brain is mainly mediated by two distinct glial cell types, astrocytes, and microglia [52] Amyloid beta (Aβ) and amyloid precursor protein (APP) activate release cytokines from microglia, astrocytes, and neurons and also promote the expression and deposition of amyloid beta [53]. An important factor in the onset of the inflammatory process is interleukin-1 (IL-1), which produces many reactions that cause dysfunction and neuronal death. Other important cytokines in neuroinflammation are interleukin-6 (IL-6) and tumor necrosis factor alfa (TNF-α). Other cytokines such as IL-1 receptor antagonist (IL-1ra), interleukins IL-4, IL-10, and transforming growth factor beta (TGF-β) have positive action and can suppress both pro-inflammatory cytokine production protecting the brain [54,55].

The recognition of an inflammatory process in the pathogenesis of neurodegenerative disease triggered the investigation of the potential use of NSAIDs in the prevention and treatment of Alzheimer’s disease (AD, Parkinson’s disease (PD, Huntington’s disease (HD, multiple sclerosis (MS and amyotrophic lateral sclerosis (ALS. Molecular and cellular potential targets were selected for pre-clinical and clinical studies to prove the therapeutic function of NSAIDs in the management of neurodegeneration diseases [42,56,57,58]. Recent studies also confirmed that ion channels, matrix metalloproteases, and microRNAs have an important place in the pathogenesis of neuroinflammation, in particular, microRNA-32 regulates microglia-mediated neuroinflammation and neurodegeneration [59].

### 3.2. Clinical Evidence

Evidence from the epidemiological observations confirmed that subjects with arthritis have a reduced incidence of AD [60]. Systematic review and meta-analysis of observational studies published between 1966 and 2002 that examined the role of NSAID use in preventing Alzheimer’s disease identified that the long-term use of NSAIDs may protect against Alzheimer’s disease but not against vascular dementia [61]. A large Alzheimer’s Disease Anti-inflammatory Prevention Trial (ADAPT) reported that the use of naproxen or celecoxib did not improve cognitive function [62]. NSAIDs have an adverse effect in later stages of AD pathogenesis, whereas asymptomatic individuals treated with conventional NSAIDs such as naproxen experience reduced AD incidence, but only after 2 to 3 years. Thus, naproxen appeared thereafter to be protective in subjects who had been asymptomatic at baseline, but treatment effects differ at various stages of the disease and that timing and choice of specific NSAID might be a key factor [63]. It should be noted that the ADAPT trial was not designed to evaluate cardiovascular events and this is in contrast with the available safety data about the cardiovascular risk of naproxen use [63].

Many further trials with different NSAIDs including indomethacin [64], ibuprofen [65], diclofenac [66], nimesulide [67], rofecoxib [68], triflusal [69], flurbiprofen [70] in patients with established AD showed no or small benefit and clinical development of novel NSAID-derived γ-secretase modulator tarenflurbil were terminated in view unsatisfactory findings [71].

The failure of the trials may be attributed to many facts, including the choice of NSAIDs and the disease stage. NSAIDs may be beneficial only in the initial suppression of Aβ deposition, microglial activation, and release of pro-inflammatory mediators at very early stages of the AD process. When the Aβ deposition process is already started, NSAIDs are no longer effective and may even be detrimental because of their inhibitory activity on chronically activated microglia that in the long-term may mediate Aβ clearance [71]. NSAID differs by pharmacokinetics and pharmacodynamics profiles, including NSAID concentration reaching the brain and COX-2-specific molecular targets, and only a subset of NSAIDs can lower Aβ production [72,73].

The short duration of the trials, choice of NSAIDs and treatment timing (patients too old or too severely ill), and the genetic variability of the patients may all have contributed to the failures. It would be helpful in the future to determine whether patients involved in trials experience changes in biomarkers in blood or CSF (such as Aβ levels, tau, or inflammatory markers) and whether those correlate with cognitive performance [74].

Long-term use of NSAIDs is associated with a reduced incidence of AD in epidemiologic studies, but randomized controlled trials with various NSAIDs including indomethacin, naproxen, celecoxib, diclofenac, and nimesulide have not replicated these findings. Thus, NSAID use cannot currently be recommended either for primary prevention or treatment of AD. However, the available evidence does suggest that cognitively normal patients taking long-term courses of NSAIDs for other indications likely have a decreased risk of AD, which represents an important finding given the high prevalence of NSAID use among older adults [75].

To date, the therapeutic paradigm for Alzheimer’s disease has focused on a single intervention for all patients. However, a new modern concept of disease pharmacotherapy supports an integrating approach into pathogenesis evaluation [76] and the precision medicine therapy model. Integrated inflammatory-based (NSAID-general and NSAID-specific) diagnostics tools have significant potential to identify select patients with AD who have a high likelihood of responding to NSAID therapy and it might be a new toll of successful clinical trials in the future [77]. Whatever the explanations for past NSAID trial failures are, based on compelling new genetic evidence for a causal role for innate immunity in AD risk, new trials with both longer and earlier interventions and alternative approaches to favorably modulate neuroinflammation are warranted [76].

### 3.3. Parkinson Disease

Investigation from experimental models and samples of PD patients suggested central and peripheral inflammatory responses of neuron and glial cells in PD pathogenesis [75]. Neuroinflammatory responses could be regulated by neuron-glia interaction which can be considered as one of the biomarkers of the PD disease diagnosis, pathogenesis, and therapeutics [78]. Neuroinflammatory response is associated with an increased level of COX and accordingly inflammation modulator Prostaglandin F2α (PGF2α) [79,80]. Studies confirmed evidence for a major role of microglial crosstalk with astrocytes, mDA neurons, and neural stem progenitor cells (NSCs) in the 1-methyl-4-phenyl-1,2,3,6-tetrahydropyridine (MPTP) mouse model of PD, and identified Wnt/β-catenin signaling, a pivotal morphogen for mDA neurodevelopment, neuroprotection, and neuroinflammatory modulation, as a critical actor in glia-neuron and glia-NSCs crosstalk [81]. Activated microglia release various factors involved in neuroinflammation, such as cytokines, chemokines, growth factors, reactive oxygen species (ROS), reactive nitrogen species (RNS), and PGs. Activated microglia interact with other cell types (e.g., neurons, astrocytes, and mast cells) and are closely associated with α-synuclein (α-syn) pathophysiology and iron homeostasis disturbance. Microglial activation and microglia-mediated inflammatory responses play essential roles in the pathogenesis of PD and elucidation of the complexity and imbalance of microglial activation may shed light on novel therapeutic approaches for PD [82].

NSAIDs were clinically used for PD patients’ treatment [83,84]. Effect of indomethacin, ibuprofen, and celecoxib on various disease-related signaling factors and mechanisms involving nitrosative stress, neurotransmission, neuronal communication, and peroxisome proliferator-activated receptor-γ has been documented in experimental PD models [85,86]. There may be a protective effect of non-aspirin NSAIDs use on the risk of PD consistent with a possible neuroinflammatory pathway in PD pathogenesis [87], but no association or week was found between regular use of various NSAIDs including aspirin and ibuprofen and reduction of PD risk from epidemiological studies [88,89,90,91,92,93]. Case-control analysis of 22,007 male aged 40–84 years without indications for or contraindications to regular NSAID use and free of Parkinson’s disease at baseline did not find evidence that NSAID use reduces PD risk. The positive associations observed between NSAID use and PD might have been due to confounding by indication as the use was clustered in the few years before disease diagnosis [94].

A recent meta-analysis of fifteen eligible studies confirmed, that NSAIDs use was not associated with the risk of Parkinson’s disease, and the potency and the cumulative NSAIDs use did not play critical roles [95,96] and clinicians have to use NSAIDs only to their approved anti-inflammatory and analgesic effects [97].

### 3.4. Amyotrophic Lateral Sclerosis

Amyotrophic lateral sclerosis, a neurodegenerative disease, causes neuronal losses in the CNS and inflammatory process is involved in the pathogenesis of the disease [98,99]. The molecular and cellular changes leading to neurodegeneration occur in the astrocytes and glial cells [100]. Administration of NSAIDs with peroxisome proliferator-activated receptor-γ (PPAR γ) agonism such as sulindac, celecoxib, rofecoxib, and nimesulide has been shown to delay motor impairment in addition to treatment with COX inhibitors and therefore may be considered as promising in the treatment of ALS and other neurodegenerative diseases [42].

### 3.5. Clinical Evidence

Data from studies showed that aspirin use might reduce the risk of ALS, and the benefit might be more prominent for older people [101], but another case-control study of incident cases (*n* = 111) conducted within the Kaiser Permanente Medical Care Program of Northern California during the years 1996–2000 found no evidence that the use of ASA or other NSAIDs prevented ALS [102]. In another study evaluating five prospective cohorts, no correlation was found between the use of NSAIDs and the risk of ALS [100].

Data from meta-analysis confirmed, that the use of non-aspirin NSAIDs and acetaminophen is associated with a decreased risk of development of ALS, and these medications seem to confer neuroprotective effects, but for more convincing evidence regarding the effectiveness of aspirin, non-aspirin NSAIDs, and acetaminophen in reducing risks of ALS, more qualified prospective studies are required [103]. The weak effectiveness of NSAIDs in neurodegenerative disease management could be explained by pharmacokinetic and pharmacodynamics data. In general, NSAIDs cross the blood-brain barrier (BBB) efficiently, but the effective dose reaching the brain can be different under different neuropathological conditions, depending on BBB integrity [104] and amphiphilic nature of NSAID which allows NSAID interaction with lipid membranes, on the modulation of membrane biomechanical properties and cell signaling events [105]. NSAID doses required for PPAR-γ agonist activity are in the high micromolar range, largely exceeding those required for in vivo inhibition of COXs. Aspirin and paracetamol possess a lack of PPAR-γ agonist activity or the activity is very weak [106] and also interindividual variations in response to NSAIDs have been reported in peripheral organs, which all makes it difficult to correlate pharmacokinetic parameters to clinical efficacy [107].

## 4. Anticancer Action

Development of nitric oxide (NO) donating aspirin formulations for the prevention of cardiovascular disease supported the pro-apoptotic [108,109,110,111], anti-proliferative [108] pro-oxidant [112], and inhibition of mitogen-activated protein kinase (MAPK) pathways [113,114] effects and possible NO donating NSAIDs application in the prevention and treatment of a variety of different cancers [115]. Studies supported the NSAIDs hypothesis, that chemoprevention of cancers such as colorectal cancer (CRC) can be either COX-dependent or COX-independent which can be synergistic at different steps of this multistep process [116] with evidence for replacement of adenomatous polyposis coli (APC) function by NSAIDs.

Studies with NSAIDs confirmed, that COX-2 selective NSAIDs might selectively inhibit the induction of apoptosis in human intestinal stem cells with aberrant Wnt signaling [117]. Aspirin reduces the risk of CRC in individuals with elevated COX-2 expression, but not in those without [118] and with associated reduced mortality [119]. Thus, these findings confirmed the involvement of prostaglandins and non-prostaglandin COX-2 products in the development of CRC [120,121,122].

The over-expression of NSAID-activated gene (NAG-1) in cancer cells results in growth arrest and an increase in apoptosis, suggesting that NAG-1 has anti-tumorigenic activity acting as a tumor suppressor in the early stages of tumor progression and the expression of NAG-1 can be increased by the COX-II inhibitors. An increase in NAG-1 is observed in inhibition of the *AKT/GSK-3 beta* pathway, suggesting NAG-1 alters cell survival [122]. Thus, forced NAG-1 expression by COX-II inhibitors could provide a mechanistic basis for the apoptotic effect of COX inhibitors in cancer cells [121,122,123,124] may serve as a potential biomarker for the diagnosis and prognosis of cancer and a therapeutic target for the inhibition and treatment of cancer development and progression [125].

Other potential anticancer action of NSAIDs can be explained by the ability of selective COX-2 inhibitors to enhance the sensitivity of lung cancer cells to NK cell-mediated cytotoxicity. Sublethal concentrations of celecoxib increased the expression levels of UL16-binding protein 1 (ULBP-1), a natural-killer group 2 member D (NKG2D) ligand, in lung cancer A549 and H460 cell lines. ULBP-1 mRNA and protein expression was induced in a dose- and time-dependent manner in lung cancer cells, thereby increasing their susceptibility to NK cell cytotoxicity. These results suggest that the effects of conventional anticancer therapy may potentially be enhanced by using celecoxib to enhance the sensitivity of lung cancer cells to NK cell-mediated cytotoxicity [126] and a combination of NSAIDs with docosahexaenoic acid (DHA), commonly derived from fish oils, would possibly synergize their anticancer activity and which can be further developed for chemoprevention and adjunct therapy in lung cancer [127].

The potential COX-2-independent mechanism of NSAIDs’ antineoplastic action includes downregulation of proto-oncogenes, such as c-Myc, and transcriptional factors such as peroxisome proliferator-activated receptor delta (PPARδ), nuclear factor kappa-light-chain-enhancer of activated B cells (NF-κB), prostate apoptosis response-4 (PAR-4), and B-cell lymphoma 2 (Bcl-2) [128,129]. Sulindac and indomethacin in vivo studies demonstrated inhibition of tumorigenesis through inhibition of peroxisome proliferator-activated receptor delta (PPARδ), a gene that is normally regulated by APC [130]. Studies have shown that NSAIDs display anticarcinogenic and chemopreventive properties through the regulation of autophagy in certain types of cancer [131]. In recent years, an increasing number of studies have indicated that NSAIDs, such as celecoxib, meloxicam, sulindac, aspirin, sildenafil, rofecoxib, and sodium salicylate, have diverse effects in cancer that are mediated by the autophagy pathway. These NSAIDs can modulate tumor autophagy through the *PI3K/Akt/mTOR, MAPK/ERK1/2, P53/DRAM, AMPK/mTOR, Bip/GRP78, CHOP/GADD153,* and *HGF/MET* signaling pathways and inhibit lysosome function, leading to p53-dependent G1 cell-cycle arrest [45,132,133,134,135,136,137,138].

### Clinical Evidence

Several epidemiologic studies have evaluated the association between the use of NSAIDs and certain types of cancer [138,139,140]. In a case-control study of 417 prostate cancer patients and 420 group-matched control subjects regular daily use of over the counter and prescription NSAIDs, ibuprofen or aspirin, was associated with a 66% reduction in prostate cancer risk accordingly odds ratio = 0.34, (95% confidence interval = 0.23–0.58, *p* < 0.01) and 0.35, (95% confidence interval = 0.15–0.84, *p* < 0.05) [141]. Another data from 91 epidemiologic studies showed a significant decline in the risk of malignancies with the regular use of NSAIDs. Daily intake of NSAIDs, primarily aspirin, produced risk reductions of 63% for colon, 39% for breast, 36% for lung, 39% for prostate, 73% for esophageal, 62% for stomach, and 47% for ovarian cancer. NSAID effects became apparent after five or more years of use, were stronger with longer-term use and cancer-protective effects were stronger for gastrointestinal malignancies (esophagus, stomach, and colon). Initial epidemiologic studies of malignant melanoma, Hodgkin’s disease, and adult leukemia also found that NSAIDs are protective, but results for pancreatic, urinary bladder, and renal cancer were inconsistent [141].

Despite evidence that NSAIDs could theoretically have anticancer properties and data from epidemiological studies that NSAIDs use can decline the risk for malignancies results from large cohort studies of NSAIDs and breast cancer (BC) risk are inconsistent [142,143,144]. French E3N prospective cohort study, which included 98,995 women did not differ by NSAID names and selectivity to COX-1 and COX-2, BC subtypes, risk factors, and comorbidities, nor by duration and dose of use. However, a statistically significant decreased risk of BC with NSAID use was only observed among women who also used PPI before [145].

The Third National Health and Nutrition Examination Study (NHANES III) data revealed that regular use of ibuprofen resulted in a 48% reduced risk of lung cancer mortality (HR = 0.52, 95% CI: 0.33–0.82, *p* < 0.01), but the main effects of other NSAIDs used, such as aspirin or acetaminophen, were not statistically significant. Thus, study results suggest that high-risk subgroups of smokers may benefit from the regular use of specific NSAIDs, which may prove to be a useful strategy for lung cancer prevention [146].

It is well known that the antitumor effects of NSAIDs mainly are related to their autophagy modulating effects, but the effectiveness of NSAIDs anti-cancer autophagy may depend on many factors of tumor and the NSAID used. The type of tumor, stage of tumorigenesis, tumor microenvironment, genetic, epigenetic factors, NSAIDs pharmacokinetics profile, and selectivity of COX-I and COX-II inhibition may have an impact on anticancer activity. Thus, further studies are warranted with the discovery of new NSAIDs anticancer mechanisms and the development of molecular biology techniques to study autophagy and understanding the effects of NSAIDs and their antitumor effects at the molecular and cellular levels.

## 5. Cardio Effects

It is widely accepted that immune activation may trigger the atherosclerotic process and that inflammation may have a potential role in the progression of atherosclerosis. Cyclooxygenases (COX) mediate the production of eicosanoids, which are involved in atherosclerotic processes in the vessel wall and platelet aggregation [147]. The production of vasoconstrictor and platelets aggregator Thromboxane A2 (TXA_2_) is mainly regulated by COX-I, while both COX-1 and COX-II are involved in the production of vasodilator and platelet aggregation inhibitor Prostacyclin (PGI_2_) [148]. It was evidence that COX-2 promotes the development of atheromatous lesions in low-density lipoprotein receptor-deficient (LDLR−/−) mice in vivo [149] and that selective inhibition of the COX-2 enzyme with celecoxib prevented the development of atherosclerotic lesions in the proximal aortas from apo E−/− mice [147]. Further studies confirmed that increased COX-2, IL-6, and matrix metalloproteinase 9 (MMP-9) levels are associated with acute ischemic syndromes [150,151,152].

These findings support the hypothesis that the COX-2/prostaglandin E2 axis may have a potential role in atherosclerosis development and its selective inhibition might be an attractive therapeutic target in atherosclerosis patients. At the very beginning COX-2 inhibitors held a promise however, clinical studies raised several clinically relevant questions as to their beneficial role in atherosclerosis prevention, because of increased thrombogenicity and cardiovascular risk, and therefore COX-2 inhibitors should be restricted in atherosclerosis patients [153]. Selective COX-2 inhibitors in all dosages and nonselective NSAIDs in high dosages increase mortality in patients with previous MI and should therefore be used with particular caution in these patients [154]. Naproxen and low dose ibuprofen (<1200 mg/day) are considered to have the most favorable thrombotic cardiovascular safety profile of all NSAIDs and is typically recommended as first line analgesics.

### Clinical Evidence

Despite results from a few studies that treatment with COX-2 inhibitors as adjuvants could be beneficial for the patients with stable angina pectoris scheduled for percutaneous coronary intervention or in patients with coronary artery disease (CAD) undergoing coronary stenting [155,156] in terms of the incidence of myocardial infarction, defined as the elevation of creatine kinase muscle type (CK-MB) [157] and less frequent reduction in the revascularization rate [158], the researchers concluded that there is still a lack of evidence regarding the long-term safety of the NSAIDs [159] or the evidence is negative [160].

Several theories have been proposed to explain the pathogenesis of atherosclerosis and in the particular inflammatory response, however, the detailed mechanisms of inflammation in atherosclerosis development have not been fully clarified, and effective diagnostic and therapeutic approaches remain limited. Studies demonstrated that the expression of miR-16 was downregulated in the cell and animal models of atherosclerosis, as the main contributor to CAD, thus these findings suggest, that the miR-16 gene from miR-16 microRNA precursor’s family may be a potential diagnostic biomarker and therapeutic target for atherosclerosis anti-inflammatory therapy [161,162].

## 6. Diabetes

There is evidence, that type 2 diabetes (T2DM) is associated with a mild-to-moderate inflammation and that has been proposed as a link to disease progression as well as its complications [163]. An increased level of IL-6 has been noted in obese individuals and in patients with T2DM [164]. Data from studies confirm, that NSAIDs could improve in vivo glucose and lipid homeostasis, and could lead to a hypothesis targeting inflammation and NF-κB as a therapeutic approach in type 2 diabetes [165]. The hypothesis that subacute-chronic inflammation contributes to the pathogenesis of obesity-related dysglycemia and that targeting inflammation may provide a therapeutic route for diabetes prevention [166] and chronic diabetic wound healing. Previous studies confirmed that diabetic wounds are trapped in a persistent inflammatory state with elevated levels of pro-inflammatory cytokines and proteases together with impaired expression of growth factors [167] and a macrophage are the primary producers of pro-inflammatory cytokines in wounds [168,169,170]. Thus, investigating the effects of NSAIDs medications on wound healing process may allow clinicians the opportunity to offer personalized diabetic patients treatments that both treat the systemic diabetic condition and chronic wounds healing [171].

## 7. Conclusions

Anti-inflammatory, antipyretic, and analgesic properties of NSAIDs are well evaluated, but many other different molecular and cellular factors which are not yet fully understood play an important role in the pathogenesis of inflammation, axonal damage, demyelination, atherosclerosis, carcinogenesis, and other pathological conditions. NSAIDs are a heterogeneous group of medicines with relative different pharmacokinetics and pharmacodynamics profiles, including inhibition of arachidonic metabolism via cyclooxygenases and lipoxygenases pathway, thus further studies based on precise NSAIDs pharmacotherapy model are warranted for the discovery of new potential NSAIDs mechanisms. Data from new studies at the molecular and cellular levels will fill in the gap between experimental and clinical results and translate our knowledge into successful disease therapy.

## Data Availability

3rd Party Data. Figure 1 and Figure 2 were obtained from Mc Graw Hill and are available at: Figure 1. Citation: Chapter 37 Lipid-Derived Autacoids: Eicosanoids and Platelet-Activating Factor, Brunton LL, Hilal-Dandan R, Knollmann BC. *Goodman & Gilman**’s: The Pharmacological Basis of Therapeutics, 13e;* 2017. Available online: https://accesspharmacy.mhmedical.com/content.aspx?bookid=2189&sectionid=170271821 accessed on: 2 April 2021 Copyright © 2021 McGraw-Hill Education. All rights reserved. Figure 2. Citation: Chapter 37 Lipid-Derived Autacoids: Eicosanoids and Platelet-Activating Factor, Brunton LL, Hilal-Dandan R, Knollmann BC. *Goodman & Gilman**’s: The Pharmacological Basis of Therapeutics, 13e;* 2017. Available online: https://accesspharmacy.mhmedical.com/content.aspx?bookid=2189&sectionid=170271821 accessed on: 2 April 2021 Copyright © 2021 McGraw-Hill Education. All rights reserved with the permission of Mc Graw Hill. Additional license agreement is signed and enclosed.

## Abbreviations

15(*S*)-hydroperoxyeicosatetraenoic acid	(15(*S*)-HpETE)
1-methyl-4-phenyl-1,2,3,6-tetrahydropyridine	(MPTP)
78-kDa glucose-regulated protein	(GRP78)
Adenomatous polyposis coli	(APC)
Adenosine triphosphate	(ATP)
AMP-activated protein kinase	(AMPK)
Amyloid beta	(Aβ)
Amyloid precursor protein	(APP)
Amyotrophic lateral sclerosis	(ALS)
Arachidonate 5-lipoxygenase	(5-LOX)
Arachidonic acid	(AA)
Binding immunoglobulin protein	(BiP)
Blood brain barrier	(BBB)
Breast cancer	(BC)
C/EBP homologous protein	(CHOP)
CCAAT/enhancer-binding proteins	(C/EBPs)
Colorectal cancer	(CRC)
Coronary artery disease	(CAD)
Damage-regulated autophagy modulator	(DRAM)
DNA damage-inducible gene 153	(GADD153)
Docosahexaenoic acid	(DHA)
Extracellular signal-regulated kinase	(ERK)
Hepatocyte growth factor	(HGF)
IL-1 receptor antagonist	(IL-1ra)
Interleukin-1	(IL-1)
Interleukin-6	(IL-6)
Creatine kinase muscle type	(CK-MB)
Large family of hydrolase enzymes that bind to the nucleotide guanosine triphosphate	(Rho-GTPases)
Lipoxins	(LXs)
Low-density lipoprotein receptor deficient	(LDLR−/−)
Matrix metalloproteinase 9	(MMP-9)
Met tyrosine kinase	(TK)
Mitogen-activated protein kinase	(MAPK)
Natural-killer group 2 member D	(NKG2D)
Neural stem progenitor cells	(NSCs)
Nitric oxide	(NO)
Non-steroidal anti-inflammatory drugs	(NSAIDs)
NSAID activated gene	(NAG-1)
Nuclear factor kappa-light-chain-enhancer of activated B cells	(NF-κB)
Parkinson Disease	(PD)
Peroxisome proliferator-activated receptor delta	(PPARδ)
Peroxisome proliferator-activated receptor-γ	(PPAR γ)
Prostacyclin	(PGI_2_)
Prostaglandin F_2α_	(PGF_2α_)
Prostaglandins	(PGs)
Reactive nitrogen species	(RNS)
Reactive oxygen species	(ROS)
Slow reacting substance in anaphylaxis	(SRS-A)
The mammalian target of rapamycin	(mTOR)
The phosphatidylinositol-3-kinase	(PI3K)/Akt
Thromboxane	(TxA_2_)
Tumor necrosis factor-alfa	(TNF-α)
Tumor protein	(P53)
Type 2 diabetes	(T2DM)
UL16-binding protein 1	(ULBP-1)
α-synuclein	(α-syn)

## References

[1] Vane J.R. (2000). The fight against rheumatism: From willow bark to COX-1 sparing drugs. J. Physiol. Pharmacol..

[2] Stone E. (1763). XXXII. An account of the success of the bark of the willow in the cure of agues. In a letter to the Right Honourable George Earl of Macclesfield, President of RS from the Rev. Mr. Edward Stone, of Chipping-Norton in Oxfordshire. Phil. Trans. R. Soc..

[3] Sneader W. (2000). The discovery of aspirin: A reappraisal. Br. Med. J..

[4] Prescott L. (1979). The third Lilly Prize Lecture. University of London, January, 1979. The nephrotoxicity and hepatotoxicity of antipyretic analgesics. Br. J. Clin. Pharmacol..

[5] Wright V. (1993). Historical overview of NSAIDs. Eur. J. Rheumatol. Inflamm..

[6] Pasero G., Marson P. (2011). A short history of anti-rheumatic therapy-V. Analgesics. Reumatismo.

[7] Vane J.R. (1971). Inhibition of prostaglandin synthesis as a mechanism of action for aspirin-like drugs. Nat. New Biol..

[8] Botting R.M. (2010). Vane’s discovery of the mechanism of action of aspirin changed our understanding of its clinical pharmacology. Pharmacol. Rep..

[9] Shampo M.A., Kyle R.A., Steensma D. (2013). John Robert Vane—British Pharmacologist and Nobel Laureate. Mayo Clin. Proc..

[10] Vane J.R., Botting R.M. (1998). Anti-Inflammatory Drugs and Their Mechanism of Action. Inflamm. Res..

[11] Coles L.S., Fries J.F., Kraines R.G., Roth S.H. (1983). From experiment to experience: Side effects of nonsteroidal anti-inflammatory drugs. Am. J. Med..

[12] Fries J. (1996). Toward an understanding of NSAID-related adverse events: The contribution of longitudinal data. Scand. J. Rheumatol..

[13] Änggård E., Samuelsson B. (1965). Biosynthesis of Prostaglandins from Arachidonic Acid in Guinea Pig Lung: Prostaglandins and related factors 38. J. Biol. Chem..

[14] Ferreira S.H., Moncada S., Vane J.R. (1974). Prostaglandins and the mechanism of analgesia produced by aspirin like drugs. Ann. R. Coll. Surg. Engl..

[15] Moncada S., Ferreira S.H., Vane J.R. (1978). Bioassay of prostaglandins and biologically active substances derived from arachidonic acid. Adv. Prostaglandin Thromboxane Res..

[16] Moncada S., Ferreira S.H., Vane J.R. (1975). Inhibition of prostaglandin biosynthesis as the mechanism of analgesia of aspirin-like drugs in the dog knee joint. Eur. J. Pharmacol..

[17] Ferreira S.H., Moncada S., Vane J.R. (1971). Indomethacin and aspirin abolish prostaglandin release from the spleen. Nat. New Biol..

[18] Higgs G.A., Mugridge K.G., Moncada S., Vane J.R. (1984). Inhibition of tissue damage by the arachidonate lipoxygenase inhibitor BW755C. Proc. Natl. Acad. Sci. USA.

[19] Ricciotti E., Fitzgerald G.A. (2011). Prostaglandins and inflammation. Arterioscler. Thromb. Vasc. Biol..

[20] Blain H., Jouzeau J.Y., Netter P., Jeandel C. (2000). Les anti-inflammatoires non steroidiens inhibiteurs selectifs de la cyclooxygenase 2. Interet et perspectives. Rev. Med. Interne.

[21] Brune K., Hinz B. (2004). The discovery and development of antiinflammatory drugs. Arthritis Rheum..

[22] Seaver B., Smith J.R. (2004). Inhibition of COX isoforms by nutraceuticals. J. Herb. Pharmacother..

[23] Atchison J.W., Herndon C.M., Rusie E. (2013). NSAIDs for musculoskeletal pain management: Current perspectives and novel strategies to improve safety. J. Manag. Care Pharm..

[24] Bannwarth B. (2000). Gastrointestinal tolerance of nonsteroidal anti-inflammatory Agents. Drugs.

[25] Singh G. (1998). Recent considerations in nonsteroidal anti-inflammatory drug gastropathy. Am. J. Med..

[26] Kendall B.J., Peura D.A. (1993). NSAID-associated gastrointestinal damage and the elderly. GI disease in the elderly series: Article five in the series. Pract. Gastroenterol..

[27] Committee on Safety of Medicines (1986). Non-steroidal anti-inflammatory drugs and serious gastrointestinal adverse reactions. Br. Med. J..

[28] Schlegel S.I., Paulus H.E., Furst D.E., Dromgoole S.H. (1987). General characteristics of nonsteroidal anti-inflammatory drugs. Drugs for Rheumatic Disease.

[29] Kowalski M.L., Woessner K., Sanak M. (2015). Approaches to the diagnosis and management of patients with a history of nonsteroidal anti-inflammatory drug-related urticaria and angioedema. J. Allergy Clin. Immunol..

[30] Praveen Rao P.N., Knaus E.E. (2008). Evolution of nonsteroidal anti-inflammatory drugs (NSAIDs): Cyclooxygenase (COX) inhibition and beyond. J. Pharm. Pharm. Sci..

[31] Simmons D.L., Botting R.M., Hla T. (2004). Cyclooxygenase isozymes: The biology of prostaglandin synthesis and inhibition. Pharmacol. Rev..

[32] Smyth E.M., Grosser T., Wang M., Yu Y., FitzGerald G.A. (2009). Prostanoids in health and disease. J. Lipid Res..

[33] Gilston V., Blake D.R., Winyard P.G. (2000). Inflammatory mediators, free radicals and gene transcription. Free Radic. Inflamm..

[34] Arend W.P., Dayer J.-M. (1995). Inhibition of the production and effects of interleukins-1 and tumor necrosis factor α in rheumatoid arthritis. Arthritis Rheum..

[35] Lone A.M., Taskén K. (2013). Proinflammatory and immunoregulatory roles of eicosanoids in T cells. Front. Immunol..

[36] Leone S., Ottani A., Bertolini A. (2007). Dual Acting Anti-Inflammatory Drugs. Curr. Top. Med. Chem..

[37] Bertolini A., Ottani A., Sandrini M. (2005). Selective COX-2 Inhibitors and Dual Acting Anti-inflammatory Drugs: Critical Remarks. Curr. Med. Chem..

[38] Fiorucci S., Meli R., Bucci M., Cirino G. (2001). Dual inhibitors of cyclooxygenase and 5-lipoxygenase. A new avenue in anti-inflammatory therapy?. Biochem. Pharmacol..

[39] Celotti F., Durand T. (2003). The metabolic effects of inhibitors of 5-lipoxygenase and of cyclooxygenase 1 and 2 are an advancement in the efficacy and safety of anti-inflammatory therapy. Prostaglandins Other Lipid Mediat..

[40] Celotti F., Laufer S. (2001). Anti-inflammatory drugs: New multitarget compounds to face an old problem. The dual inhibition concept. Pharmacol. Res..

[41] Ling Q.L., Murdoch E., Ruan K.H. (2016). How can we address the controversies surrounding the use of NSAIDS in neurodegeneration?. Future Med. Chem..

[42] Lleo A., Galea E., Sastre M. (2007). Molecular targets of non-steroidal anti-inflammatory drugs in neurodegenerative diseases. Cell Mol. Life Sci..

[43] Chatterjee K., Jana S., Choudhary P., Swarnakar S. (2018). Triumph and tumult of matrix metalloproteinases and their crosstalk with eicosanoids in cancer. Cancer Metastasis Rev..

[44] Chang C.Y., Li J.R., Wu C.C., Wang J.D., Liao S.L., Chen W.Y., Chen C.J. (2020). Endoplasmic reticulum stress contributes to indomethacin-induced glioma apoptosis. Int. J. Mol. Sci..

[45] Yu C., Li W.B., Liu J.B., Lu J.W., Feng J.F. (2018). Autophagy: Novel applications of nonsteroidal anti-inflammatory drugs for primary cancer. Cancer Med..

[46] Ciotu C.I., Fischer M.J.M. (2020). Novel Analgesics with Peripheral Targets. Neurotherapeutics.

[47] Shadfar S., Hwang C.J., Lim M.S., Choi D.Y., Hong J.T. (2015). Involvement of inflammation in Alzheimer’s disease pathogenesis and therapeutic potential of anti-inflammatory agents. Arch. Pharmacal. Res..

[48] Ahmad M.H., Fatima M., Mondal A.C. (2019). Influence of microglia and astrocyte activation in the neuroinflammatory pathogenesis of Alzheimer’s disease: Rational insights for the therapeutic approaches. J. Clin. Neurosci..

[49] Park J.C., Han S.H., Mook-Jung I. (2020). Peripheral inflammatory biomarkers in Alzheimer’s disease: A brief review. BMB Rep..

[50] Sang C.N., Schmidt W.K. (2020). Aligning New Approaches to Accelerate the Development of Non-opioid Analgesic Therapies. Neurotherapeutics.

[51] Chen W.W., Zhang X., Huang W.J. (2016). Role of neuroinflammation in neurodegenerative diseases (Review). Mol. Med. Rep..

[52] Jha M.K., Jeon S., Suk K. (2012). Glia as a Link between Neuroinflammation and Neuropathic Pain. Immune Netw..

[53] Solfrizzi V., D’Introno A., Colacicco A.M., Capurso S.A., Pietrarossa G., Santamato V., Capurso A.P.F. (2006). Circulating biomarkers of cognitive decline and dementia. Clin. Chim. Acta.

[54] Rubio-Perez J.M., Morillas-Ruiz J.M. (2012). A review: Inflammatory process in Alzheimer’s disease, role of cytokines. Sci. World J..

[55] Small D.H., Klaver D.W., Foa L. (2010). Presenilins and the γ-secretase: Still a complex problem. Mol. Brain.

[56] Ransohoff R.M. (2016). How neuroinflammation contributes to neurodegeneration. Science.

[57] Ray R., Juranek J.K., Rai V. (2016). RAGE axis in neuroinflammation, neurodegeneration and its emerging role in the pathogenesis of amyotrophic lateral sclerosis. Neurosci. Biobehav. Rev..

[58] Subhramanyam C.S., Wang C., Hu Q., Dheen S.T. (2019). Microglia-mediated neuroinflammation in neurodegenerative diseases. Semin. Cell Dev. Biol..

[59] Niranjan R. (2018). Recent advances in the mechanisms of neuroinflammation and their roles in neurodegeneration. Neurochem. Int..

[60] Mcgeer P.L., Mcgeer E., Rogers J., Sibley J. (1990). Anti-inflammatory drugs and Alzheimer disease. Lancet.

[61] Veld T.B.A., Ruitenberg A., Hofman A., Launer L.J., van Duijn C.M., Stijnen T. (2001). Nonsteroidal Antiinflammatory Drugs and the Risk of Alzheimer’s Disease. N. Engl. J. Med..

[62] Martin B.K., Szekely C., Brandt J., Piantadosi S., Breitner J.C.S., Craft S., Evans D., Green R. (2008). Cognitive function over time in the Alzheimer’s disease anti-inflammatory prevention trial (ADAPT): Results of a randomized, controlled trial of naproxen and celecoxib. Arch. Neurol..

[63] Breitner J.C., Baker L.D., Montine T.J., Meinert C.L., Lyketsos C.G., Ashe K.H., Brandt J., Craft S., Evans D.E., Green R.C. (2011). Extended results of the Alzheimer’s disease anti-inflammatory prevention trial. Alzheimer’s Dement..

[64] Angiolillo D.J., Weisman S.M. (2017). Clinical Pharmacology and Cardiovascular Safety of Naproxen. Am. J. Cardiovasc. Drugs.

[65] Rogers J., Kirby L.C., Hempelman S.R., Berry D.L., McGeer P.L., Kaszniak A.W. (1993). Clinical trial of indomethacin in alzheimer’s disease. Neurology.

[66] Pasqualetti P., Bonomini C., Dal Forno G., Paulon L., Sinforiani E., Marra C., Rossini P.M. (2009). A randomized controlled study on effects of ibuprofen on cognitive progression of Alzheimer’s disease. Aging Clin. Exp. Res..

[67] Scharf S., Mander A., Ugoni A., Vajda F., Christophidis N. (1999). A double-blind, placebo-controlled trial of diclofenac/misoprostol in Alzheimer’s disease. Neurology.

[68] Aisen P.S., Schmeidler J., Pasinetti G.M. (2002). Randomized pilot study of nimesulide treatment in alzheimer’s disease. Neurology.

[69] Reines S.A., Block G.A., Morris J.C., Liu G., Nessly M.L., Lines C.R., Norman B.A., Baranak C.C. (2004). Rofecoxib: No effect on Alzheimer’s disease in a 1-year, randomized, blinded, controlled study. Neurology.

[70] Gómez-Isla T., Blesa R., Boada M., Clarimón J., Del Domenech G. (2008). A randomized, double-blind, placebo controlled-trial of triflusal in mild cognitive impairment: The TRIMCI study. Alzheimer Dis. Assoc. Disord..

[71] Mintzer J.E., Wilcock G.K., Black S.E., Zavitzk H., Hendrix S.B. (2006). MPC-7869 (R-flurbiprofen), a selective Ab42-lowering agent, delays time to clinically significant psychiatric events in Alzheimer_s disease (AD): Analysis from a 12-month phase 2 trial. Alzheimer’s Dement..

[72] Uddin S., Kabir T., Jeandet P., Mathew B., Ashraf G.M., Perveen A., Bin-Jumah M.N., Mousa S.A., Abdel-Daim M.M. (2020). Novel Anti-Alzheimer’s Therapeutic Molecules Targeting Amyloid Precursor Protein Processing. Oxidative Med. Cell. Longev..

[73] Weggen S., Eriksen J., Das P., Sagi S.A., Wang R., Pietrzik C.U., Findlay K.A., Smith T.E., Murphy M.P., Bulter T. (2001). A subset of NSAIDs lower amyloidogenic Aβ42 independently of cyclooxygenase activity. Nat. Cell Biol..

[74] Sastre M., Dewachter I., Landreth G.E., Willson T.M., Klockgether T., Van Leuven F., Heneka M.T. (2003). Nonsteroidal Anti-Inflammatory Drugs and Peroxisome Proliferator-Activated Receptor-γ Agonists Modulate Immunostimulated Processing of Amyloid Precursor Protein through Regulation of β-Secretase. J. Neurosci..

[75] Sastre M., Gentleman S.M. (2010). NSAIDs: How they work and their prospects as therapeutics in Alzheimer’s disease. Front. Aging Neurosci..

[76] Yang L., Mao K., Yu H., Chen J. (2020). Neuroinflammatory Responses and Parkinson’ Disease: Pathogenic Mechanisms and Therapeutic Targets. J. Neuroimmune Pharmacol..

[77] Heneka M.T., Carson M.J., El Khoury J., E Landreth G., Brosseron F., Feinstein D.L., Jacobs A.H., Wyss-Coray T., Vitorica J., Ransohoff R.M. (2015). Neuroinflammation in Alzheimer’s disease. Lancet Neurol..

[78] O’Bryant S.E., Zhang F., Johnson L., Hall J., Edwards M., Grammas P., Oh E., Lyketsos C.G., Rissman R.A. (2018). A Precision Medicine Model for Targeted NSAID Therapy in Alzheimer’s Disease. J. Alzheimer’s Dis..

[79] Panicker N., Saminathan H., Jin H., Neal M., Harischandra D.S., Gordon R., Kanthasamy K., Lawana V., Sarkar S., Luo J. (2015). Fyn Kinase Regulates Microglial Neuroinflammatory Responses in Cell Culture and Animal Models of Parkinson’s Disease. J. Neurosci..

[80] Pasinetti G.M. (1998). Cyclooxygenase and inflammation in Alzheimer’s disease: Experimental approaches and clinical interventions. J. Neurosci. Res..

[81] Corwin C., Nikolopoulou A., Pan A.L., Nunez-Santos M., Vallabhajosula S., Serrano P. (2018). Prostaglandin D2/J2 signaling pathway in a rat model of neuroinflammation displaying progressive parkinsonian-like pathology: Potential novel therapeutic targets 11 Medical and Health Sciences 1109 Neurosciences. J. Neuroinflammation.

[82] L’Episcopo F., Tirolo C., Serapide M.F., Caniglia S., Testa N., Leggio L., Vivarelli S., Iraci N., Pluchino S., Marchetti B. (2018). Microglia Polarization, Gene-Environment Interactions and Wnt/β-Catenin Signaling: Emerging Roles of Glia-Neuron and Glia-Stem/Neuroprogenitor Crosstalk for Dopaminergic Neurorestoration in Aged Parkinsonian Brain. Front. Aging Neurosci..

[83] Liu C.Y., Wang X., Liu C., Zhang H.L. (2019). Pharmacological Targeting of Microglial Activation: New Therapeutic Approach. Front. Cell. Neurosci..

[84] Moore A.H., Bigbee M.J., Boynton G.E., Wakeham C.M., Rosenheim H.M., Staral C.J., Morrissey J.L., Hund A.K. (2010). Non-Steroidal Anti-Inflammatory Drugs in Alzheimer’s Disease and Parkinson’s Disease: Reconsidering the Role of Neuroinflammation. Pharm..

[85] Klegeris A., McGeer P.L. (2005). Non-steroidal anti-inflammatory drugs (NSAIDs) and other anti-inflammatory agents in the treatment of neurodegenerative disease. Curr. Alzheimer Res..

[86] Singh A., Tripathi P., Singh S. (2020). Neuroinflammatory responses in Parkinson’s disease: Relevance of Ibuprofen in therapeutics. Inflammopharmacology.

[87] Ramazani E., Tayarani-Najaran Z., Fereidoni M. (2019). Celecoxib, indomethacin and ibuprofen prevent 6-hydroxydopamine-induced PC12 cell death through the inhibition of NFκB and SAPK/JNK pathways. Iran. J. Basic Med. Sci..

[88] Gagne J.J., Power M.C. (2010). Anti-inflammatory drugs and risk of Parkinson disease: A meta-analysis. Neurology.

[89] Chen H., Jacobs E., Schwarzschild M.A., McCullough M.L., Calle E.E., Thun M.J., Ascherio A. (2005). Nonsteroidal antiinflammatory drug use and the risk for Parkinson’s disease. Ann. Neurol..

[90] Etminan M., Suissa S. (2008). NSAID Use and the Risk of Parkinsons Disease. Curr. Drug Saf..

[91] Hernán M.A., Logroscino G., Rodríguez L.A.G. (2006). Nonsteroidal anti-inflammatory drugs and the incidence of Parkinson disease. Neurology.

[92] Bower J.H., Maraganore D.M., Peterson B.J., Ahlskog J.E., Rocca W.A. (2006). Immunologic diseases, anti-inflammatory drugs, and Parkinson disease: A case-control study. Neurology.

[93] Becker C., Jick S.S., Meier C.R. (2011). NSAID use and risk of Parkinson disease: A population-based case-control study. Eur. J. Neurol..

[94] Manthripragada A.D., Schernhammer E., Qiu J., Friis S., Wermuth L., Olsen J.H., Ritz B. (2011). Non-Steroidal Anti-Inflammatory Drug Use and the Risk of Parkinson’s Disease. Neuroepidemiology.

[95] Driver J.A., Logroscino G., Lu L., Gaziano J.M., Kurth T. (2011). Use of non-steroidal anti-inflammatory drugs and risk of Parkinson’s disease: Nested case-control study. BMJ.

[96] Ren L., Yi J., Yang J., Li P., Cheng X., Mao P. (2018). Nonsteroidal anti-inflammatory drugs use and risk of Parkinson disease: A dose–response meta-analysis. Medicine.

[97] Etminan M., Carleton B.C., Samii A. (2008). Non-steroidal anti-inflammatory drug use and the risk of Parkinson disease: A retrospective cohort study. J. Clin. Neurosci..

[98] Poly T.N., Islam M.M., Yang H.C., Li Y.C.J. (2019). Non-steroidal anti-inflammatory drugs and risk of Parkinson’s disease in the elderly population: A meta-analysis. Eur. J. Clin. Pharmacol..

[99] Agius L.M. (2012). Neuroinflammation as the proximate cause of signature pathogenic pattern progression in amyotrophic lateral sclerosis, aids, and multiple sclerosis. Pathol. Res. Int..

[100] Lee J., Hyeon S.J., Im H., Ryu H., Kim Y., Ryu H. (2016). Astrocytes and microglia as non-cell autonomous players in the pathogenesis of ALS. Exp. Neurobiol..

[101] Fondell E., O’Reilly E.J., Fitzgerald K.C., Falcone G.J., McCullough M.L., Thun M.J., Park Y., Kolonel L.N., Ascherio A. (2012). Non-steroidal anti-inflammatory drugs and amyotrophic lateral sclerosis: Results from five prospective cohort studies. Amyotroph. Lateral Scler..

[102] Tsai C.P., Lin F.C., Lee J.K.W., Lee C.T.C. (2015). Aspirin use associated with amyotrophic lateral sclerosis: A total population-based case-control study. J. Epidemiol..

[103] Popat R.A., Tanner C.M., Eeden S.K.V.D., Bernstein A.L., Bloch D.A., Leimpeter A., McGuire V., Nelson L.M. (2007). Effect of non-steroidal anti-inflammatory medications on the risk of amyotrophic lateral sclerosis. Amyotroph. Lateral Scler..

[104] Chang M.C., Kwak S.G., Park J.S., Park D. (2020). The effectiveness of nonsteroidal anti-inflammatory drugs and acetaminophen in reduce the risk of amyotrophic lateral sclerosis? A meta-analysis. Sci. Rep..

[105] Parepally J.M., Mandula H., Smith Q.R. (2006). Brain uptake of nonsteroidal anti-inflammatory drugs: Ibuprofen, flurbiprofen, and indomethacin. Pharm. Res..

[106] Zhou Y., Dial E.J., Doyen R., Lichtenberger L.M. (2010). Effect of indomethacin on bile acid-phospholipid interactions: Implication for small intestinal injury induced by nonsteroidal anti-inflammatory drugs. Am. J. Physiol. Gastrointest. Liver Physiol..

[107] Ajmone-Cat M.A., Bernardo A., Greco A., Minghetti L. (2010). Non-steroidal anti-inflammatory drugs and brain inflammation: Effects on microglial functions. Pharmaceuticals.

[108] Day R.O., Brooks P.M. (1987). Variations in response to non-steroidal anti-inflammatory drugs. Br. J. Clin. Pharmacol..

[109] Huguenin S., Vacherot F., Kheuang L., Fleury-Feith J., Jaurand M.-C., Bolla M., Riffaud J.-P., Chopin M.K. (2004). Antiproliferative effect of nitrosulindac (NCX 1102), a new nitric oxide-donating non-steroidal anti-inflammatory drug, on human bladder carcinoma cell lines. Mol. Cancer Ther..

[110] Gebril S.M., Ito Y., Shibata M., Maemura K., Abu-Dief E.E., Hussein M.R.A., Abdelaal U.M., Elsayed H.M., Otsuki Y., Higuchi K. (2020). Indomethacin can induce cell death in rat gastric parietal cells through alteration of some apoptosis- and autophagy-associated molecules. Int. J. Exp. Pathol..

[111] Fecker L.F., Stockfleth E., Nindl I., Ulrich C., Forschner T., Eberle J. (2007). The role of apoptosis in therapy and prophylaxis of epithelial tumours by nonsteroidal anti-inflammatory drugs (NSAIDs). Br. J. Dermatol..

[112] Chiou S.K., Hoa N., Hodges A. (2011). Sulindac sulfide induces autophagic death in gastric epithelial cells via Survivin down-regulation: A mechanism of NSAIDs-induced gastric injury. Biochem. Pharmacol..

[113] Gao J., Liu X., Rigas B. (2005). Nitric oxide-donating aspirin induces apoptosis in human colon cancer cells through induction of oxidative stress. Proc. Natl. Acad. Sci. USA.

[114] Hundley T.R., Rigas B. (2006). Nitric oxide-donating aspirin inhibits colon cancer cell growth via mitogen-activated protein kinase activation. J. Pharmacol. Exp. Ther..

[115] Hynes J., Leftheris K. (2005). Small Molecule p38 Inhibitors: Novel Structural Features and Advances from 2002–2005. Curr. Top. Med. Chem..

[116] Kashfi K., Rigas B. (2005). Non-COX-2 targets and cancer: Expanding the molecular target repertoire of chemoprevention. Biochem. Pharmacol..

[117] Rigas B., Shiff S.J. (1999). Nonsteroidal anti-inflammatory drugs and the induction of apoptosis in colon cells: Evidence for PHS-dependent and PHS-independent mechanisms. Apoptosis.

[118] Wang D., Dubois R.N. (2010). The role of COX-2 in intestinal inflammation and colorectal cancer. Oncogene.

[119] Qiu W., Wang X., Leibowitz B., Liu H., Barker N., Okada H., Oue N., Yasui W., Clevers H., Schoen R.E. (2010). Chemoprevention by nonsteroidal anti-inflammatory drugs eliminates oncogenic intestinal stem cells via SMAC-dependent apoptosis. Proc. Natl. Acad. Sci. USA.

[120] Chan A.T., Ogino S., Fuchs C.S. (2007). Aspirin and the Risk of Colorectal Cancer in Relation to the Expression of COX-2. N. Engl. J. Med..

[121] Fischer S., Hawk E., Lubet R. (2011). Non-steroidal anti-inflammatory drugs and coxibs in chemoprevention: A commentary based primarily on animal studies. Cancer Prev. Res..

[122] Liggett J.L., Zhang X., Eling T.E., Baek S.J. (2014). Anti-tumor activity of non-steroidal anti-inflammatory drugs: Cyclooxygenase-independent targets. Cancer Lett..

[123] Eling T.E., Baek S.J., Shim M., Lee C.H. (2006). NSAID activated gene (NAG-1), a modulator of tumorigenesis. J. Biochem. Mol. Biol..

[124] Jang T.J., Kang H.J., Kim J.R., Yang C.H. (2004). Non-steroidal anti-inflammatory drug activated gene (NAG-1) expression is closely related to death receptor-4 and -5 induction, which may explain sulindac sulfide induced gastric cancer cell apoptosis. Carcinogenesis.

[125] Jang T.J., Kim N.I., Lee C.H. (2006). Proapoptotic activity of NAG-1 is cell type specific and not related to COX-2 expression. Apoptosis.

[126] Wang X., Baek S.J., Eling T.E. (2013). The diverse roles of nonsteroidal anti-inflammatory drug activated gene (NAG-1/GDF15) in cancer. Biochem. Pharmacol..

[127] Kim J., Noh M.H., Hur D.Y., Kim B., Kim Y.S., Lee H.K. (2020). Celecoxib upregulates ULBP 1 expression in lung cancer cells via the JNK/PI3K signaling pathway and increases susceptibility to natural killer cell cytotoxicity. Oncol. Lett..

[128] Poku R.A., Jones K.J., van Baren M., Alan J.K., Amissah F. (2020). Diclofenac enhances docosahexaenoic acid-induced apoptosis in vitro in lung cancer cells. Cancers.

[129] Duran A., Linares J.F., Galvez A.S., Wikenheiser K., Flores J.M., Diaz-Meco M.T., Moscat J. (2008). The Signaling Adaptor p62 Is an Important NF-κB Mediator in Tumorigenesis. Cancer Cell.

[130] Luo S., Rubinsztein D.C. (2007). Atg5 and Bcl-2 provide novel insights into the interplay between apoptosis and autophagy. Cell Death Differ..

[131] He T.C., Chan T.A., Vogelstein B., Kinzler K.W. (1999). PPARδ is an APC-regulated target of nonsteroidal anti-inflammatory drugs. Cell.

[132] Liu M., Li C.-M., Chen Z.-F., Ji R., Guo Q.-H., Li Q., Zhang H.-L., Zhou Y.-N. (2014). Celecoxib regulates apoptosis and autophagy via the PI3K/Akt signaling pathway in SGC-7901 gastric cancer cells. Int. J. Mol. Med..

[133] Kaneko S., Kaneko M., Fukushima T. (2013). Enhanced antitumor effect of lower-dose and longer-term CPT-11 treatment in combination with low-dose celecoxib against neuroblastoma xenografts. Int. J. Clin. Oncol..

[134] Crighton D., Wilkinson S., O’Prey J., Syed N., Smith P., Harrison P.R., Gasco M., Garrone O., Crook T., Ryan K.M. (2006). DRAM, a p53-Induced Modulator of Autophagy, Is Critical for Apoptosis. Cell.

[135] Johnson A.J., Hsu A.L., Lin H.P., Song X., Chen C.S. (2002). The cyclo-oxygenase-2 inhibitor celecoxib perturbs intracellular calcium by inhibiting endoplasmic reticulum Ca2+-ATPases: A plausible link with its anti-tumour effect and cardiovascular risks. Biochem. J..

[136] Kroemer G., Mariño G., Levine B. (2010). Autophagy and the Integrated Stress Response. Mol. Cell.

[137] Kim Y.C., Guan K.L. (2015). MTOR: A pharmacologic target for autophagy regulation. J. Clin. Investig..

[138] Chapuis N., Tamburini J., Green A.S., Willems L., Bardet V., Park S., Lacombe C., Mayeux P., Bouscary D. (2010). Perspectives on inhibiting mTOR as a future treatment strategy for hematological malignancies. Leukemia.

[139] Nelson J.E., Harris R.E. (2000). Inverse association of prostate cancer and non-steroidal anti-inflammatory drugs (NSAIDs): Results of a case-control study. Oncol. Rep..

[140] Harris R.E. (2009). Cyclooxygenase-2 (cox-2) blockade in the chemoprevention of cancers of the colon, breast, prostate, and lung. Inflammopharmacology.

[141] Harris R. (2001). Inverse association of non-steroidal anti-inflammatory drugs and malignant melanoma among women. Oncol. Rep..

[142] Harris R.E., Beebe-Donk J., Doss H., Burr Doss D. (2005). Aspirin, ibuprofen, and other non-steroidal anti-inflammatory drugs in cancer prevention: A critical review of non-selective COX-2 blockade (review). Oncol. Rep..

[143] Clarke C.A., Canchola A.J., Moy L.M., Neuhausen S.L., Chung N.T., Jr J.V.L., Bernstein L. (2017). Regular and low-dose aspirin, other non-steroidal anti-inflammatory medications and prospective risk of HER2-defined breast cancer: The California Teachers Study. Breast Cancer Res..

[144] Marshall S.F., Bernstein L., Anton-Culver H., Deapen D., Horn-Ross P.L., Mohrenweiser H., Peel D., Pinder R., Purdie D.M., Reynolds P. (2005). Nonsteroidal Anti-Inflammatory Drug Use and Breast Cancer Risk by Stage and Hormone Receptor Status. J. Natl. Cancer Inst..

[145] Gill J.K., Maskarinec G., Wilkens L.R., Pike M.C., Henderson B.E., Kolonel L.N. (2007). Nonsteroidal Antiinflammatory Drugs and Breast Cancer Risk: The Multiethnic Cohort. Am. J. Epidemiol..

[146] Cairat M., al Rahmoun M., Gunter M.J., Severi G., Dossus L., Fournier A. (2020). Use of nonsteroidal anti-inflammatory drugs and breast cancer risk in a prospective cohort of postmenopausal women. Breast Cancer Res..

[147] Bittoni M.A., Carbone D.P., Harris R.E. (2017). Ibuprofen and fatal lung cancer: A brief report of the prospective results from the Third National Health and Nutrition Examination Survey (NHANES III). Mol. Clin. Oncol..

[148] Jacob S., Laury-Kleintop L., Lanza-Jacoby S. (2008). The Select Cyclooxygenase-2 Inhibitor Celecoxib Reduced the Extent of Atherosclerosis in Apo E−/− Mice. J. Surg. Res..

[149] Catella-Lawson F., Reilly M., Kapoor S.C., Cucchiara A.J., Demarco S., Tournier B., Vyas S.N., Fitzgerald G.A. (2001). Cyclooxygenase Inhibitors and the Antiplatelet Effects of Aspirin. New Engl. J. Med..

[150] Burleigh M.E., Babaev V.R., Oates J.A., Harris R.C., Gautam S., Riendeau D., Marnett L.J., Morrow J.D., Fazio S., Linton M.F. (2002). Cyclooxygenase-2 Promotes Early Atherosclerotic Lesion Formation in LDL Receptor–Deficient Mice. Circ..

[151] Cipollone F., Prontera C., Pini B., Marini M., Fazia M., De Cesare D., Iezzi A., Ucchino S., Boccoli G., Saba V. (2001). Overexpression of Functionally Coupled Cyclooxygenase-2 and Prostaglandin E Synthase in Symptomatic Atherosclerotic Plaques as a Basis of Prostaglandin E 2 -Dependent Plaque Instability. Circ..

[152] Zhao S.-P., Deng P., Huang H.-G., Xu Z.-M., Dai H.-Y., Hong S.-C., Yang J., Zhou H.-N. (2005). Expression of COX-2 mRNA in Peripheral Blood Monocytes from Patients with Acute Myocardial Infarction and Its Significance. Clin. Chem..

[153] Cuccurullo C., Mezzetti A., Cipollone F. (2007). COX-2 and the vasculature: Angel of evil?. Curr. Hypertens. Rep..

[154] Press Release European Medicines Agency Concludes Action on COX-2 Inhibitors. http://www.emea.eu.int.

[155] Gislason G.H., Jacobsen S., Rasmussen J.N., Rasmussen S., Buch P., Friberg J., Schramm T.K., Abildstrom S.Z., Køber L., Madsen M. (2006). Risk of Death or Reinfarction Associated With the Use of Selective Cyclooxygenase-2 Inhibitors and Nonselective Nonsteroidal Antiinflammatory Drugs After Acute Myocardial Infarction. Circulation.

[156] Ozdol C., Gulec S., Rahimov U., Atmaca Y., Turhan S., Erol C. (2007). Naproxen treatment prevents periprocedural inflammatory response but not myocardial injury after percutaneous coronary intervention. Thromb. Res..

[157] Saadeddin S.M. (2003). Percutaneous coronary intervention in the context of systemic inflammation: More injury and worse outcome. Med. Sci. Monit..

[158] Pelliccia F., Pasceri V., Granatelli A., Pristipino C., Speciale G., Roncella A., Cianfrocca C., Mercuro G., Richichi G. (2006). Safety and Efficacy of Short-Term Celecoxib Before Elective Percutaneous Coronary Intervention for Stable Angina Pectoris. Am. J. Cardiol..

[159] Koo B.-K., Kim Y.-S., Park K.-W., Yang H.-M., Kwon D.-A., Chung J.-W., Hahn J.-Y., Lee H.-Y., Park J.-S., Kang H.-J. (2007). Effect of celecoxib on restenosis after coronary angioplasty with a Taxus stent (COREA-TAXUS trial): An open-label randomised controlled study. Lancet.

[160] Chung J.W., Yang H.M., Park K.W., Lee H.Y., Park J.S., Kang H.J. (2010). Long-term outcome of adjunctive celecoxib treatment after paclitaxel-eluting stent implantation for the complex coronary lesions: Two-year clinical follow-up of COREA-TAXUS trial. Circulation.

[161] Bally M., Beauchamp M.E., Abrahamowicz M., Nadeau L., Brophy J.M. (2018). Risk of acute myocardial infarction with real-world NSAIDs depends on dose and timing of exposure. Pharmacoepidemiol. Drug Saf..

[162] Wang M., Li J., Cai J., Cheng L., Wang X., Xu P., Li G., Liang X. (2020). Overexpression of MicroRNA-16 Alleviates Atherosclerosis by Inhibition of Inflammatory Pathways. BioMed Res. Int..

[163] Zhang Y.H., He K., Shi G. (2017). Effects of MicroRNA-499 On the Inflammatory Damage of Endothelial Cells during Coronary Artery Disease Via the Targeting of PDCD4 Through the NF-Κβ/TNF-α Signaling Pathway. Cell. Physiol. Biochem..

[164] Schmidt M.I., Duncan B.B., Sharrett A.R., Lindberg G., Savage P.J., Offenbacher S., Azambuja M.I., Tracy R.P., Heiss G. (1999). Markers of inflammation and prediction of diabetes mellitus in adults (Atherosclerosis Risk in Communities study): A cohort study. Lancet.

[165] Pradhan A.D., Manson J.E., Rifai N., Buring J.E., Ridker P.M. (2001). C-reactive protein, interleukin 6, and risk of developing type 2 diabetes mellitus. J. Am. Med. Assoc..

[166] Goldfine A.B., Silver R., Aldhahi W., Cai D., Tatro E., Lee J., Shoelson S.E. (2008). Use of Salsalate to Target Inflammation in the Treatment of Insulin Resistance and Type 2 Diabetes. Clin. Transl. Sci..

[167] Fleischman A., Shoelson S.E., Bernier R., Goldfine A.B. (2008). Salsalate improves glycemia and inflammatory parameters in obese young adults. Diabetes Care.

[168] Eming S.A., Martin P., Tomic-Canic M. (2014). Wound repair and regeneration: Mechanisms, signaling, and translation. Sci. Transl. Med..

[169] Mirza R.E., Fang M.M., Ennis W.J., Kohl T.J. (2013). Blocking interleukin-1β induces a healing-associated wound macrophage phenotype and improves healing in type 2 diabetes. Diabetes.

[170] Mirza R.E., Fang M.M., Novak M.L., Urao N., Sui A., Ennis W.J., Koh T.J. (2015). Macrophage PPARγ and impaired wound healing in type 2 diabetes. J. Pathol..

[171] Salazar J.J., Ennis W.J., Koh T.J. (2016). Diabetes medications: Impact on inflammation and wound healing. J. Diabetes Complicat..

